# First Japanese case of maternal phenylketonuria treated with sapropterin dihydrochloride and the normal growth and development of the child

**DOI:** 10.1016/j.ymgmr.2019.100526

**Published:** 2019-11-01

**Authors:** Hiromi Nyuzuki, Taro Yamazaki, Megumi Saito, Akira Ohtake

**Affiliations:** a Department of Pediatrics, Niigata University, Niigata, Japan; b Division of Functional Genomics & Systems Medicine, Research Center for Genomic Medicine, Saitama Medical University, Saitama, Japan; c Department of Pediatrics, Saitama Medical University, Saitama, Japan; d Department of Clinical Genomics, Saitama Medical University, Saitama, Japan

**Keywords:** Maternal phenylketonuria, Sapropterin dihydrochloride, Growth and development

## Abstract

Sapropterin dihydrochloride (SD) may be a new treatment option for women with phenylketonuria (PKU) who plan to become pregnant. We report the first Japanese case of maternal PKU treated with SD. The patient was administered SD at 10–20 mg/kg/day, which increased phenylalanine tolerance during the pregnancy and lactation. No adverse events occurred, and she delivered a healthy neonate. Normal growth and development of the child confirms the efficacy and safety of SD.

## 1 Introduction

Phenylketonuria (PKU) is an inherited disorder of metabolism that is associated with a deficient phenylalanine (Phe) hydroxylating system, resulting in elevated plasma levels of Phe. Phenylalanine hydroxylase (PAH) is a key enzyme that catalyzes the conversion of Phe to tyrosine (Tyr). High plasma Phe levels during pregnancy are teratogenic [1]. Birth defects associated with maternal PKU include facial dysmorphism, congenital heart disease, intrauterine growth retardation, microcephaly, and developmental delay [2]. Although maternal plasma Phe concentrations prior to conception must be strictly controlled to prevent the development of the aforementioned abnormalities in the neonate, it is often difficult to re-establish strict dietary management to a sufficient degree in women with PKU [3]. Tetrahydrobiopterin (BH4) is a cofactor of PAH, and pharmacological doses of exogenous BH4 are known to increase the stability of PAH to prevent its degradation [4]. Recent studies have demonstrated the safety and efficacy of sapropterin dihydrochloride (SD), a synthetic form of BH4, in cases of maternal PKU. Low-dose SD treatment in cases of maternal PKU was first reported by Koch et al. in 2005 [5]. In 2014, a European study reported the safety and efficacy of SD at pharmacological doses in 8 cases of maternal PKU [2]. Pharmacological doses of SD may be viewed as a new option for women with PKU desiring pregnancy. However, to date, no report has described neonatal developmental outcomes after birth to establish the safety of SD use during pregnancy and lactation. We report the first Japanese case of maternal PKU treated with SD with normal growth and development observed in the child born to the woman.

## 2 Case report

We describe a woman who was diagnosed with PKU based on newborn screening. Her Phe level in a dried blood spot was 360–480 μmol/L based on the Guthrie method. Her plasma Phe level was 960–1200 μmol/L during the neonatal period, and she was instructed to consume a low-Phe diet. Her metabolic status was well controlled, as her Phe tolerance changed with age: 60–80 mg/kg/day at 0–1 year(s), 40–60 mg/kg/day at 1–3 years, 30–40 mg/kg/day at >3 years, and 20–30 mg/kg/day (1000–1500 mg/day) in adulthood. Genetic testing revealed a *PAH* mutation (R241C/Ivs4nt-1 g > a), which was consistent with her phenotype of mild PKU.

She desired to become pregnant at the age of 28 years and initiated strict dietary management. She reduced her daily Phe intake from 1500 mg/day to 200–400 mg/day and used Phe-free formula. Her plasma Phe level quantified based on liquid chromatography/mass spectrometry reduced from a mean of 519 μmol/L (before strict dietary management) to 155 μmol/L (after strict dietary management), and she continued strict dietary management for 2 years. Unfortunately, she was unable to conceive, discontinued strict dietary management, and abandoned the plan for pregnancy. Her daily Phe intake increased to 1500 mg/day, and she discontinued taking Phe-free formula.

**Fig. 1:**
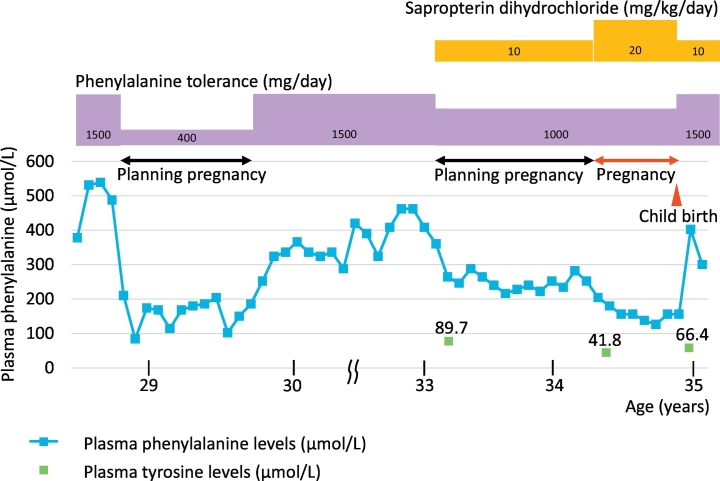
The clinical course of the patient and the efficaty of SD treatment.

Subsequently, at 32 years of age, she decided to retry to become pregnant; however, strict dietary management was difficult. A BH4 loading test at a dose of 20 mg/kg for 7 days was performed prior to pregnancy. She showed a clear response, and her plasma Phe level reduced from 529 μmol/L to 285 μmol/L (rate of decrease was 46.1%). Owing to the difficulty with re-establishing strict dietary therapy, oral administration of SD was initiated in preparation for pregnancy beginning at 33 years of age. The administration was halted during the menstrual period owing to its high cost in Japan. SD was administered at a dose of 10 mg/kg body weight (daily dosage of 500 mg) in combination with a Phe-reduced diet, which restricted nutritional Phe intake to 1000 mg/day. As the restriction was milder than before, she was able to continue the required dietary management. The mean plasma Phe level was 248 μmol/L (range 216–288 μmol/L). She conceived following treatment for a year, and the SD dose was increased to 20 mg/kg body weight (daily dosage of 1000 mg) at 6 weeks of gestation to optimize metabolic control. The patient's treatment course is shown in Fig. 1. Her plasma Phe levels were maintained below 300 μmol/L, and Tyr levels were in the normal range (40.4–90.3 μmol/L) during preconception and pregnancy on SD treatment without any additional nutritional supplements. No SD treatment-induced adverse event was observed, and she maintained herself in good condition during her pregnancy. Prenatal ultrasonography revealed normal fetal development. She delivered a healthy male neonate at 39 weeks and 4 days of gestation (birth weight, height, and head circumference of 3498 g (1.34 standard deviation), 50.1 cm (0.51 standard deviation), and 34.0 cm (0.53 standard deviation), respectively). The neonate passed newborn screening. She continued SD treatment after childbirth, reducing the SD dose to 10 mg/kg body weight (daily dosage of 500 mg). Breastfeeding on SD treatment was continued until the child was 25 months old. The Phe tolerance was increased to 1000–1500 mg/day with SD treatment during the lactation period.

The neonate achieved normal developmental milestones. Evaluation performed at 31 months of age using the Enjoji Developmental Scale [6] showed that his developmental quotients for physical movements, hand movements, basic daily habits, personal relations, speaking, and understanding of language were 121, 109, 133, 109, 109, and 121, respectively. His weight, height, and head circumference at 43 months of age were 16.0 kg (0.71 standard deviation), 96.0 cm (−0.39 standard deviation), and 49.8 cm (−0.05 standard deviation), respectively.

## 3 Discussion

We reported here the first Japanese case of maternal PKU treated with SD. As the plasma Phe level before treatment was 960–1200 μmol/L, the phenotype in our patient was classified as mild PKU according to the severity of PKU [7]. Plasma Phe levels were well controlled in our patient with the administration of SD along with a Phe-reduced diet throughout preconception, pregnancy, and lactation. The Phe tolerance was increased from 400 mg/day (before SD treatment) to 1000–1500 mg/day (during SD treatment). SD use was not associated with any adverse events, and excellent outcomes were observed in the child.

Strict control of plasma Phe levels is warranted in women with PKU who plan to conceive and in those who do become pregnant to prevent birth defects associated with maternal PKU. Although the plasma Phe levels in our patient before pregnancy were controlled within the adult target range (120–600 μmol/L), women with PKU who are planning to conceive and are pregnant are recommended to control the plasma Phe levels within 120–360 μmol/L [8], which requires additional treatment (strict dietary management or SD treatment). Treatment is recommended even prior to conception because previous studies have shown significant differences in general developmental status and intelligence scores in neonates born to women with PKU who are categorized into pre- and post-conception Phe-restricted dietary groups [9,10]. In contrast, Teissier et al. reported that compared with PKU during pregnancy, low plasma Phe levels (<120 μmol/L) and Tyr deficiency are associated with intrauterine growth retardation [11]. As SD ameliorates the residual enzyme activity of PAH by increasing the stability of the PAH protein [4], SD use in cases of maternal PKU is expected to improve the nutritional status, increasing protein intake and Tyr synthesis, which is associated with improved intrauterine growth and favorable neonatal outcomes. Furthermore, protein demand increases not only during pregnancy, but also during the lactation period for maternal health and fetal, neonatal, and infantile growth. As SD improves Phe tolerance in cases of PKU in non-pregnant and pregnant women, SD treatment not only helps patients with PKU continue their diet, but it also greatly helps the children's growth throughout the pregnant and lactation periods [12,13].

Genotype–phenotype correlations in PKU are well described to date [14,15]. Our patient harbored R241C/Ivs4nt-1 g > a compound heterozygous mutations in *PAH*, both of which are relatively frequent mutations in Japan (R241C: 10.1%, Ivs4nt-1 g > a: 8.9%) [15]. While Ivs4nt-1 g > a is suspected to correlate with the classical PKU phenotype, the R241C mutated allele showed residual 10% normal *in vitro* PAH activity, which is associated with the mild PKU and mild hyperphenylalaninemia phenotype in Japanese patients and is known to be responsive to treatment with BH4 [15].

Sakamoto et al. reported the first successful case of maternal PKU treated with SD [16]; however, they did not report neonatal neurodevelopmental outcomes. The Enjoji Developmental Scale is widely used in Japan to assess motor and psychological development in children. This scale consists of exercise (physical and hand movements), sociality (basic daily habits and personal relations), and language (speaking and understanding of language) scores. In this case, the child showed normal growth and development, confirming the safety of SD use throughout pregnancy and lactation period. Our case confirms the efficacy and safety of SD administration in pregnant women with PKU. Further studies are warranted to confirm the effectiveness of pharmacological doses of SD to treat women with PKU who plan to become pregnant.
